# Safety perception and determinants of complementary and alternative medicine usage among surgery out-patients in LAUTECH Teaching hospital, ogbomoso, Nigeria

**DOI:** 10.1016/j.heliyon.2024.e24835

**Published:** 2024-01-20

**Authors:** Oluwatosin Stephen Ilori, Olawale Olakunlehin, Oluwatosin Ruth Ilori, Phillip Oluwatobi Awodutire, Chidi Ugwuoke, Olajumoke Shittu

**Affiliations:** a Department of Surgery, Ladoke Akintola University of Technology Teaching Hospital, Nigeria; b Department of Community Medicine, Ladoke Akintola University of Technology Teaching Hospital, Nigeria; c Department of Quantitative Health Science, Cleveland Clinic, USA

**Keywords:** Complementary and alternative medicine, Homeopathic medicine, Surgery outpatients

## Abstract

**Objectives:**

The use of complementary and alternative medicine (CAM) stemmed from the search of humans for other means of relieving pain and managing diseases which has plagued its existence. CAM use is on the increase among the general population in both the developed and developing nations and also among surgical patients. There is therefore a need to ascertain the perceived adverse effects, the safety perception and the determinants of its use so as to improve the advocacy for adequate regulation.

**Methods:**

It was a cross-sectional study carried out among surgical outpatients in a tertiary hospital. One hundred and fifty patients between the ages of 18 and 85 years were recruited. An interviewer-administered questionnaire was used to collect data from each participant. Data was analyzed using SPSS version 22.

**Results:**

The lifetime prevalence of CAM use among the respondents was 76 % while the point/current prevalence was 37.3 %. The percentage of current users using CAM for surgical complaints was 30.4 %. Biological based therapy accounted for 110 (72 %) of CAM used and unbranded herbal products was responsible for more than two-third of it. Almost a third of the patients (46, 30.7 %) perceived that CAM is safe while 62 (41.3 %) were not sure of its safety. Only 15 (13.2 %) and 6 (5.3 %) have ever recorded side effects and drug interactions respectively. Older age group, income less than 10,000 Naira, positive safety perception and belief about CAM were identified as determinants of CAM usage.

**Conclusions:**

The prevalence of CAM usage among surgical outpatients was quite high and the major determinants of its use are the patient's age, safety perception and their level of income.

## 1 Introduction

The use of complementary and alternative medicine (CAM) stemmed from the search of humans for other means of relieving pain and managing diseases which has plagued its existence. This has also been guided by their various beliefs, values and cultural background [1]. CAM includes a wide range of products and practices used to manage surgical or medical conditions, but not regarded as part of conventional medicine [2]. It can be further broken down into Complementary medicine and Alternative medicine. Complementary medicine is the care system that is applied together with and in addition to medical care; while alternative medicine includes the care which take the place of medical treatment [3]. In short, CAM can serve either as a complementary or alternative therapy based on the patient's usage, whether they are combined with conventional medicine or not. Some of the therapeutic products and practices employed include traditional medicine, herbal medicine, acupuncture, bone setting etcetera.

The use of CAM is on the increase among the general population in both the developed and developing nations. It is commonly used in conditions such as fever, headache, neck and back pain, insomnia etc [4]. Globally, the prevalence of CAM use ranges from 30 to 75 % [5]. In some other studies, higher prevalence of CAM usage have been reported in the North Western (84 %) [6] and South Western (90 %) part of Nigeria [7]. In Africa, CAM has been known to encompass local herbal medicines, indigenous health care practice like traditional bone setting and the use of imported CAM products and practices like acupuncture and chiropractic [8]. The use of traditional herbs and remedies are also well known and common in Nigeria [9].

Majority of CAM users employ it as an adjunct to orthodox medicine to prevent diseases [10]. They also appreciate other features of CAM that are not typical of orthodox medicine like relatively lower cost, attention to patient's personal needs and cultural sensitivity [11]. The factors that determine CAM use vary from one place to the other and they include: age, educational status, prognosis of the disease condition and the level of income. In the developed world, the use of CAM is commoner among females of high socioeconomic status; young adults/middle aged individuals and people with higher educational attainment [9]. In the developing world, however, the determining factors are diverse.

Surgical patients’ reasons for using CAM are also diverse. It has been broadly used in patients with non-life threatening ailments as well as in life threatening conditions like cancers [12]. According to Ang-Lee et al. [13], surgical patients are likely to use herbal medications more often than the general population especially during the peri-operative period. The prevalence of herbal medication use of 22 %, 32 % and 30 % respectively have been quoted in different studies among peri-operative surgical patients [14]. Herbal medications have been known to affect the peri-operative care of surgical patients [15] by causing significant morbidity and mortality as a result of their interaction with anaesthetic drugs. Safety concerns have also been raised on the improper use of other forms of CAM among surgical patients as a result of their side effects and drug-drug interactions [12]. Some of the complications that can result from CAM use among the surgical population reported in the literature include excessive bleeding, stroke, prolonged anaesthesia etc [13].

A major factor to be considered for any study on the use of CAM is the issue of disclosure of CAM use by the patients. Kaye et al. [16] found out that 70 % of their patients were not willing to disclose their use of herbal medications. Some of the reasons adduced by Ang-Lee et al. [13] for patients' non-disclosure include physicians’ likely prejudice against the use of herbal preparations, fear of the possible consequences of admitting its use and the use of the preparations for reasons other than medical. On the other hand, the patients who talk to their surgeons about their health related beliefs and preferences for Complementary medicine often have better adherence to peri-operative instructions [12].

A review on the attitude of doctors to CAM showed that they raise a number of concerns on the efficacy, safety and the absence of appropriate regulation for such medications. In addition, there may be a need to know the degree of adverse effects associated with CAM so as to improve the advocacy for adequate policy and regulations to ensure safe and efficacious use [17]. Currently, there are limited studies on the use and safety perception of CAM among surgical patients especially those with non-malignant conditions in Nigeria. The objective of this work is therefore to determine the prevalence of CAM use among surgical outpatients, the patients’ safety perception, perceived side effects, experience with CAM-conventional drug interaction and the factors that determine its use. The surgical population was studied because there are few literature on the use of CAM among surgical patients especially in Nigeria. Also, the surgical patients are a peculiar population which may come down with deleterious effect if they experience untoward interaction between conventional drugs and CAM.

## 2 Methods

### 2.1 Setting/study population

The study was carried out among Surgery Outpatients- (Orthopaedic and trauma, Plastic Surgery, General Surgery and Urology) in LAUTECH Teaching Hospital, Ogbomoso, Oyo state, Nigeria. The recruited patients comprised adults between the ages of 18 and 85 years.

### 2.2 Study design/sampling

This was a cross-sectional study using a systematic sampling method. The patients were recruited from 31st of March till 30th of June 2021. The list of patients seen in the outpatient clinic of each of the four surgical units (Urology, General Surgery, Orthopaedic Surgery and Plastic Surgery) was retrieved from the clinical records. The average number of the out-patients seen per week in each clinic was obtained from the list. A proportional allocation of the number of patients required for each unit was done based on the calculated sample size to get the number of respondents to be sampled per unit. During every clinic day for each unit, the first patient to be seen by the consultant was selected first followed by every 5th patient. Sampling continued each clinic day until the desired size for each unit was gotten. All surgical outpatients aged between 18 and 85 years who presented with any surgical conditions (like benign prostate hyperplasia, breast cancer, thyroid enlargement, chronic leg ulcers, osteoarthritis etcetera) and who gave consent were recruited. The following patients were however excluded: patients with cognitive impairment and patients with underlying psychiatric disorders.

**2.3 Variables of interest-** The dependent outcome measure was the use of CAM while the main independent variables include the patient's belief about CAM, their safety perception, and the record of side effects. The two major groups that gave rise to the comparison were the CAM users and the non-users irrespective of the type of CAM used. Most of the comparison was based on those who were currently using versus those who were not currently using CAM. In a few instances, the comparison was between those who have ever and those who have never used CAM.

### 2.3 Sample size calculation

#### 2.3.1 Leslie fischer formula

$$n=z^{2}\text{pq}\;/\;d^{2}$$

n = the sample size required

z = standard deviate set at 1.96 (confidence interval of 95 %)

p = prevalence of CAM usage in a study (90 %) [17]

q = 1-p

d = the marginal error (0.05).

n = 138.

After adding a non-response rate of 10 %, the calculated sample size is 149.2 approximated to 150 respondents.

### 2.4 Questionnaire

An interviewer-administered questionnaire was used to collect data from each participant. The questionnaire was developed by the author from questions asked from similar studies in the literature. It was divided into five parts which include-socio-demographics, type of CAM used, pattern of CAM usage, belief about CAM and safety perception of CAM. It contained only two closed ended questions and the remaining questions were open ended. The questionnaire was pretested among 15 patients in the General Outpatient clinic before use in this study. The questionnaires were applied anonymously after explaining to each of the participants what was considered as CAM. The interviewer bias is a possible bias in this study and it was taken care of by using those who were not directly involved in the care of the patients in each unit to administer the questionnaire.

### 2.5 Ethical consideration

Ethical approval was obtained from the Ethical Review Committee of the hospital. Written informed consent was also completed by each participant who agreed to participate after the study has been explained in English or the local dialect to them. Each participant was also informed that participation was voluntary and they were assured of anonymity and confidentiality of information.

### 2.6 Data analysis

The data was analyzed using IBM SPSS version 22. Categorical variables were presented as frequencies and percentages. The Chi square test was used to compare categorical variables and multi-nominal regression model was used to determine the predictor factors. The significance level was set at 0.05.

## 3 Results

**Fig. 1:**
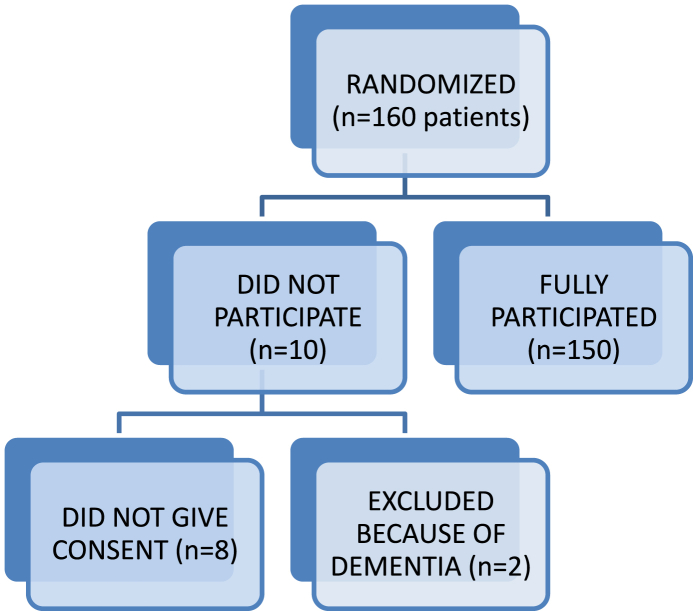
Flow chart showing patient randomization and participation..

One hundred and sixty patients (160) were randomized but only one hundred and fifty (150) eventually participated in the study. Of the ten patients that did not participate in the study, eight did not give consent while the remaining two were excluded because of dementia (see Fig. 1 below).

The result of the study showed that the largest proportion of respondents were in 41–60 years (50, 33.3 %) age bracket followed by 21–40 years (44, 29.3 %); with mean age of 48.93 ± 18.98. Males accounted for 81 (54 %) of the participants; 106 (70.7 %) were married while 36 (24 %) were single. Nearly all of the participants were of Yoruba ethnicity (147, 98.0 %). Seventy three (48.7 %) had tertiary education, while only 10 (6.7 %) had no formal education. Fifty six (37.3 %) were skilled workers while 37 (24.7 %) were unemployed. Sixty (40 %) of the participants earn between 10 and 50,000 Naira monthly while 19 (12.7 %) earn more than 100,000 Naira monthly (Table 1).

**Table 1:** Sociodemographic factors.

VARIABLES	FREQUENCY	PERCENTAGE (%)
**N** = **150**		
**Age**		
<20	9	6.0
21–40	44	29.3
41–60	50	33.3
61–80	39	26.0
>80	8	5.3
**Sex**		
Male	81	54.0
Female	69	46.0
**Marital status**		
Single	36	24.0
Married	106	70.7
Divorced	2	1.3
Widow/widower	6	4.0
**Tribe**		
Yoruba	147	98.0
Igbo	2	1.3
Others	1	0.7
**Religion**		
Christianity	103	68.7
Islam	45	30.0
Traditionalist	2	1.3
**Level of education**		
No formal	10	6.7
Primary	24	16.0
Secondary	43	28.7
Tertiary	73	48.7
**Occupation**		
Unemployed	37	24.7
Unskilled	30	20.0
Semi-skilled	27	18.0
Skilled	56	37.3
**Average monthly income (Naira)**		
<10,000	39	26.0
10-50,000	60	40.0
>50,000–100,000	32	21.3
>100,000	19	12.7

The lifetime prevalence of CAM use among the respondents was 76.0 % while the point/current prevalence was 37.3 %. (Table 2).

**Table 2:** Prevalance of CAM use (ever used and current use).

Variables	Frequency	Percentage (%)
**(N** = **150)**		
Ever used CAM		
Yes	114	76.0
No	36	24.0
**(N** = **150)**		
Current CAM use		
Yes	56	37.3
No	94	62.7

Biological based therapy accounted for 51 (91.1 %) of CAM used and unbranded herbal products were responsible for more than two-third (48, 85.7 %) of it; this was followed by spiritual therapy (43, 76.8 %), manipulative/body-based therapy (18, 32.1 %), mind-body therapy (12, 21.4 %) and whole body therapy (2, 3.6 %) in descending order (Table 3).

**Table 3:** Types of CAM currently used.

Variables	Frequency	Percentage (%)
**(N** = **56)**^a^		
**Biological based**	**51**	**91.1**
Unbranded herbal drugs	48	85.7
High dose vitamins	5	8.9
“Kedi products”	5	8.9
Medicinal tea	10	17.9
“Forever living products”	4	7.1
GNLD products	2	3.6
Other chinese products	2	3.6
Urine therapy	3	5.4
**Spiritual therapy**	**43**	**76.8**
Anointing oil	30	53.6
Prayer	34	60.7
Holy water	18	32.1
Divination and incantation	3	5.4
**Whole body therapy**	**2**	**3.6**
Acupuncture	1	1.8
Spinal manipulation	1	1.8
**Mind body therapy**	**12**	**21.4**
Meditation	4	7.1
Faith healing	10	17.9
**Manipulative/Body based therapy**	**18**	**32.1**
Massage	10	17.9
Scarification	7	12.5
Blood letting	1	1.8

a Multiple choice responses allowed.

The commonest indication for CAM use was acute infections (febrile illnesses, upper respiratory tract infection, acute diarrhea etc) and it accounted for 16 (28.6 %) of the cases. The main reason why CAM users explored its use was positive personal experience with CAM (24, 42.9 %); this was followed by search for effective treatment of diseases (13, 23.2 %) (Table 4).

**Table 4:** Indications and reasons for current CAM usage.

VARIABLES	FREQUENCY	PERCENTAGE (%)
**(N** = **114)**		
**Symptoms CAM was used for**		
Acute infections	16	28.6
Infertility/pile	11	19.6
Musculoskeletal pain	9	16.1
Non-specific symptoms	7	12.5
Fractures/Limb deformities	4	7.1
Prostate cancer/BPH	3	5.4
Leg ulcer/Osteomyelitis	3	5.4
Goitre	2	3.6
Breast cancer	1	1.7
**(N** = **114)**		
**Reason for exploring CAM**		
Perceived limitation of conventional health care	11	19.6
Dissatisfaction with pharmaceutical focus of conventional care	2	3.6
Search for effective treatment for disease	13	23.2
Positive personal experience with CAM	24	42.9
Others	6	10.7

Acute infections include: acute febrile illnesses, upper respiratory tract infections, acute diarrhea etcetera.

Seventeen (30.4 %) of the current users were making use of CAM for surgical complaints while 39 (69.6 %) used them for non-surgical complaints. More than two-third (43, 76.7 %) of the current CAM users combine CAM with orthodox medicine. A larger percentage of the CAM was used either daily (15, 26.8 %) or monthly 15 (26.8 %) and the commonest route of administration was oral (50, 74.6 %) (Table 5). Almost a third of the patients perceived that CAM was safe (46, 30.7 %) while 62, (41.3 %) were not sure of its safety. The major safety concern was lack of appropriate dosing regimen (45, 30.0 %). Out of those who have ever used CAM, only 15 (13.2 %) and 6 (5.3 %) respectively recorded side effects and drug interaction. Nearly half of the side effects experienced were non-specific (10, 43.5 %), followed by generalized body discomfort 4 (17.4 %) (Table 6).

**Table 5:** Practice of current CAM use.

VARIABLES	FREQUENCY	PERCENTAGE (%)
**(N** = **56)**		
**CurrentCAM usage complaints**		
Surgical	17	30.4
Non-surgical complaint	39	69.6
**(N** = **56)**		
**Pattern of current CAM usage**		
CAM only	13	23.2
CAM with orthodox medicine	43	76.7
**(N** = **56)**		
**Frequency of CAM use**		
Daily	15	26.8
Weekly	12	21.4
Monthly	15	26.8
Once in two month	2	3.6
Annually	4	7.1
Less than annually	6	10.7
Anytime	2	3.6
**(N** = **67)**^a^		
**Route of CAM administration**		
Oral	50	74.6
Skin application	16	23.9
Via eyes	1	1.5

a Multiple choice response allowed.

**Table 6:** Safety perception, concerns and CAM profile of respondents.

VARIABLES	FREQUENCY	PERCENTAGE (%)
**(N** = **150)**		
**Safety perception of CAM**		
Not sure	62	41.3
Safe	46	30.7
Not safe	42	28.0
**(N** = **187)**^a^		
**Safety concerns of CAM**		
Hygiene concern	40	26.7
Adverse effect	34	22.7
Lack of dosing regimen	45	30.0
Content label	24	16.0
No obvious reason	44	29.3
**(N** = **114)**		
**Record of side effect**		
Yes	15	13.2
No	81	71.1
Not sure	18	15.8
**(N** = **114)**		
**Record of interaction with orthodox drug**		
Yes	6	5.3
No	66	57.9
Not sure	42	36.8
**(N** = **23)**^a^		
**Side effect recorded**		
Not specific	10	43.5
Generalized body discomfort	4	17.4
Dizziness	3	13.0
Weakness	3	13.0
Diarrhea	2	8.7
Constipation	1	4.3

a Multiple choices allowed.

Bivariate analysis using Chi square showed that age, marital status, average monthly income and safety perception were significantly associated with CAM usage (ever used). According to Table 7 however, only safety perception was significantly associated with current CAM usage. All the statements on the patients' belief about CAM were significantly associated with CAM usage (current use) except ‘CAM are healthier than taking conventional drugs’, ‘CAM is more effective than orthodox medicine’, ‘The people who do not have money are more likely to use CAM’.. (Table 8).

**Table 7:** Association between sociodemographic characteristics, safety perception and current CAM use.

Variables	Current CAM use		TOTAL	STATISTICS
	Yes	No		
**Age (Years)**				
<20	4 (7.1)	6 (6.4)	10 (6.7)	
21-40	16 (28.6)	28 (29.8)	44 (29.3)	X^2^ = 0.857
41-60	20 (35.7)	29 (30.9)	49 (32.7)	Df = 4
61-80	14 (25.0)	25 (26.6)	39 (26.0)	Pvalue = 0.931
>80	2 (3.6)	6 (6.4)	8 (5.3)	
**Sex**				
Male	35 (62.5)	46 (48.9)	81 (54)	X^2^ = 2.599 Df = 1 Pvalue = 0.107
Female	21 (37.5)	48 (51.1)	69 (46)	
**Marital status**				
Single	14 (25.0)	22 (23.4)	36 (24)	X^2^ = 1.653 Df - 3 Pvalue = 0.647
Married	39 (69.6)	67 (71.3)	106 (70.7)	
Divorced/Separated	0 (0.0)	2 (2.1)	2 (1.3)	
Widow/Widower	3 (5.4)	3 (3.2)	6 (4.0)	
**Tribe**				
Yoruba	55 (98.2)	92 (97.9)	147 (98)	X^2^ = 2.870 Df = 2 Pvalue = 0.238
Ibo	0 (0.0)	2 (2.1)	2 (1.3)	
Others	1 (1.8)	0 (0.0)	1 (0.7)	
**Level of Education**				
No formal education	8 (5.3)	2 (1.3)	10 (6.7)	X^2^ = 1.961 Df = 1 Pvalue = 0.581
Primary	20 (13.3)	4 (2.7)	24 (16.0)	
Secondary	34 (22.7)	9 (6.0)	43 (28.7)	
Tertiary	52 (34.7)	21 (14)	73 (48.7)	
**Religion**				
Christianity	38 (67.9)	65 (69.1)	103 (68.7)	X^2^ = 1.337 Df = 2 Pvalue = 0.921
Islam	18 (32.1)	27 (28.7)	45 (30)	
Traditionalist	0 (0.0)	2 (2.1)	2 (1.3)	
**Average monthly income**				
<10,000 naira	15 (26.8)	24 (25.5)	39 (26)	X^2^ = 0.993 Df = 3 Pvalue = 0.803
10,000–50,000 naira	21 (37.5)	39 (41.5)	60 (40)	
51,000–100,000 naira	14 (25)	18 (19.1)	32 (21.3)	
>100,000 naira	6 (10.7)	13 (13.8)	19 (12.7)	
**Safety perception**				
Not sure	14 (25.0)	48 (51.1)	62 (41.3)	X^2^ = 16.947 Df = 2 Pvalue= <0.0001
Safe	28 (50.0)	18 (19.1)	46 (30.7)	
Not safe	14 (25.0)	28 (29.8)	42 (28.0)

**Table 8:** Association between belief about CAM and the current use of CAM.

Variables	Current CAM use		TOTAL	STATISTICS
	Yes	No		
**CAM is very good**				
Agree	44 (78.6)	40 (42.6)	84 (56)	X^2^ = 19.443 Df = 2 Pvalue<0.0001
Indifferent	7 (12.5)	21 (22.3)	28 (18.7)	
Disagree	5 (8.9)	33 (35.1)	38 (25.3)	
**CAM has less side effects**				
Agree	34 (60.7)	31 (33.0)	65 (43.3)	X^2^ = 10.999 Df = 2 Pvalue = 0.004
Indifferent	10 (17.9)	28 (29.8)	38 (25.3)	
Disagree	12 (21.4)	35 (37.2)	47 (31.3)	
**CAM use will increase if the government develops i**t				
Agree	38 (67.9)	43 (45.7)	81 (54.0)	X^2^ = 6.918 Df = 2 Pvalue = 0.03
Indifferent	14 (25.0)	39 (41.5)	53 (35.3)	
Disagree	4 (7.1)	12 (12.8)	16 (10.7)	
**CAM are healthier than taking conventional drugs**				
Agree	11 (19.6)	10 (10.6)	21 (14)	X^2^ = 5.150 Df = 2 Pvalue = 0.076
Indifferent	23 (41.1)	30 (31.9)	53 (35.3)	
Disagree	22 (39.3)	54 (57.4)	76 (50.7)	
**CAM enhances the body's own defensive mechanism**				
Agree	37 (66.1)	31 (33.0)	68 (45.3)	X^2^ = 16.375 Df = 2 Pvalue<0.0001
Indifferent	12 (21.4)	31 (33.0)	43 (28.7)	
Disagree	7 (12.5)	32 (34.0)	39 (26.0)	
**CAM is good for physical, spiritual and mental health**				
Agree	37 (66.1)	26 (27.7)	63 (42)	X^2^ = 23.163 Df = 2 Pvalue<0.0001
Indifferent	14 (25.0)	36 (38.3)	50 (33.3)	
Disagree	5 (8.9)	32 (34.0)	37 (24.7)	
**The more knowledge about CAM, the more the use**				
Agree	45 (80.4)	41 (43.6)	86 (57.3)	X^2^ = 20.845 Df = 2 Pvalue= <0.0001
Indifferent	9 (16.1)	30 (31.9)	39 (26.0)	
Disagree	2 (3.6)	23 (24.5)	25 (16.7)	
**The more my friends use CAM, the more likely I also use it**				
Agree	18 (32.1)	2 (1.3)	28 (18.7)	X^2^ = 10.963 Df = 2 Pvalue = 0.004
Indifferent	16 (28.6)	12 (8)	47 (31.3)	
Disagree	22 (39.3)	22 (14.7)	75 (50.0)	
**CAM is more effective than orthodox medicine**				
Agree	10 (17.9)	12 (12.8)	22 (14.7)	X^2^ = 3.616 Df = 2 Pvalue = 0.164
Indifferent	24 (42.9)	30 (31.9)	54 (36.0)	
Disagree	22 (39.3)	52 (55.3)	74 (49.3)	
**The more the fear for conventional medicine the more likely to use CAM**				
Agree	30 (53.6)	27 (28.7)	57 (38)	X^2^ = 10.143 Df = 2 Pvalue = 0.006
Indifferent	12 (21.4)	39 (41.5)	51 (34)	
Disagree	14 (25.0)	28 (29.8)	42 (28)	
**The people who do not have money are more likely to use CAM**				
Agree	34 (60.7)	46 (48.9)	80 (53.3)	X^2^ = 2.002 Df = 2 Pvalue = 0.368
Indifferent	9 (16.1)	21 (22.3)	30 (20.0)	
Disagree	13 (23.2)	27 (28.7)	40 (26.7)	

Significant values (p < 0.05).

The predictors of CAM use were determined using the multi-nominal regression model. The factors were entered into the model in forward stepwise fashion. The entry probability was 0.05 while the removal probability was 0.1 determined by likelihood ratio. According to Table 9, the older the patients the more likely they have ever used CAM. Respondents earning < N10,000 Naira and those earning N50,000 – N100,000 Naira were 8.074 and 4.753 times respectively more likely to have ever used CAM than those earning more than 100,000 Naira. Patients who had safe perception about CAM were 10.233 times more likely to have ever used CAM than those with unsafe perception. In addition, patients who had safe perception about CAM were 3.111 times more likely to be current CAM users than those with unsafe perception. (Table 9).

**Table 9:** PEDICTORS of CAM use (ever used and current use).

EVER USED CAM			
MODEL VARIABLES	β	OR (95 % CI)	Df (p-value)
**Age**			
<20	−19.064	5.255E-9 (8.271E-10-3.339E-8)	1 (<0.0001)
21–40	−18.109	1.366E-8 (3.684E-9-5.062-E8)	1 (<0.0001)
41–60	−18.044	1.457E-8 (4.023E-9-5.278E-8)	1 (<0.0001)
61–80	−16.651	5.866E-8 (5.866E-8-5.866E-8)	1
>80 (ref)	0^b^		
**Income**			
<N 10,000	2.087	8.074 (1.793–36.353)	1 (0.007)
N10,000- N50,000	1.167	3.212 (0.882–11.695)	1 (0.077)
>N50,000- N100,000	1.559	4.753 (1.094–20.653)	1 (0.038)
>100,000 (ref)	0^b^		
**Safety perception**			
Not sure	0.496	1.642 (0.647–4.166)	1 (0.297)
Safe	2.326	10.233 (2.502–41.860)	1 (0.001)
Not safe (ref)	0^b^		
X^2^ = 35.375, p-value<0.0001			
**CURRENT CAM USE**			
MODEL VARIABLE(S)	β	OR (95%CI)	Df (p-value)
**Safety perception** Not sure −0.539 Safe 1.135 Not safe (ref) 0^b^		0.583 (0.243–1.400) 3.111 (1.299–7.449)	1 (0.227) 1 (0.011)
X^2^ = 16.931, p-value<0.0001			

## 4 Discussion

The use of CAM is on the increase with variations between different populations [9,18,19]. In this current study, it was found that almost three out of four respondents have ever used CAM while every four out of ten respondents are currently using one form of CAM or the other. This is quite lower than that found among Hematology outpatients in Lagos University Teaching Hospital, where almost every nine out of ten respondents were found to have used CAM within three months preceding the study [7]. In a cross-sectional survey of three local government areas in Enugu state by Onyiapat et al., 620 (87.4 %) of the respondents acknowledged the use of CAM at some point in time [19]. The rate is however higher than that previously reported in the use of CAM by cancer patients in University Teaching Hospital (UNTH), Enugu [9]. This difference could be as a result of the variations in the disease conditions of the population group studied.

The type of CAM used by respondents in this study was consistent with the most frequently used CAM products in the literature [9,18,19]. The most commonly used form of CAM in Nigeria is herbal preparations, followed by faith healing/prayer [19]. The biological products (herbal preparation) were the most frequently used CAM products by the respondents in this study for the care of both surgical and non-surgical complaints. This is supported by findings in the United States where herbal preparations were found to be the most common form of CAM used among the elderly [20]. Spiritual therapy was the next common form of CAM therapy used by the respondents. This finding is supported by Singh et al. [18] who recorded that herbs and spiritual healing were the two most common forms of CAM used among Indians in South Africa. A study done by Tor-Anyiin et al. among health workers however showed that the most commonly used CAM was spiritual therapy followed by herbal products [21]. In this study, the unbranded herbal products accounted for the largest number of biological products used. A few other patients were also using other branded foreign products like “Kedi”, medicinal tea, “forever living products”, Golden Neo-Life Diamite (GNLD) products, high dose vitamins etc. The multi-level marketing strategies that portray these products as natural herbs and food supplements might have been responsible for their increasing popularity among the black population.

The main reasons why the surgical patients who took part in the study used CAM were for acute infections like febrile illness, upper respiratory tract infections, acute diarrhea etcetera; others include non-specific symptoms, Infertility/pile and musculoskeletal pain. For the current CAM users 30.4 % of the participants were using CAM for their surgical complaint, the remaining were using CAM for non-surgical complaints. In another study by Culha et al. [4], pain (44.6 %) accounted for the main indication for CAM use, other complaints include stress, sleeping problems and tiredness. Compared to this study, only 8.7 % of their patients used CAM to deal with problems which required surgery.

Majority of the current CAM users in this study consumed CAM products orally while very few used it as topical skin application. A large number of the current users also use them either daily or monthly while a few used them weekly. This study showed that more than three-fourth of the current CAM users combined it with conventional medicine while only slightly above one fifth used CAM only. This value is noticed to be higher than that obtained in another study in which most of the respondents did not use CAM in combination with conventional medicine, but only 40 % of them used CAM together with conventional medicine [22]. The use of CAM together with conventional medicine has however been known to lead to unanticipated reactions [23,24].

Three fifth of the respondents were not sure about the safety of CAM especially the biological products; only one third of them thought they were safe while the remaining believed they were not safe. This is in contrast to findings from a study by Jimoh et al. among a cross-section of residents in Sokoto, North-western Nigeria, where a larger fraction of the respondents (54 %) thought CAM was safe while 29 % were not sure about its safety [6] The possible reason for the higher positive safety perception recorded by Jimoh et al. might be due to the heterogeneity of the respondents and that the respondents were not being managed for any surgical or medical condition in the hospital. This perception may not be unconnected to the common ideology that CAM are mostly from natural products and hence has to be safe. The perception of CAM safety may also be due to misconception of the potential hazards of some herbal products. Of the patients who have ever used CAM in this study, only a little above one tenth have ever experienced any side effects while only 5.3 % ever recorded interaction with orthodox drugs; Jimoh et al.^6^ however had more respondents who have had serious side effects from CAM.

In this study, patients who used CAM were more likely to be of older age, persons who earn less than 100, 000 Naira monthly and those who have a positive safety perception and belief about CAM. In studies by Busari et al. [25], those with post-secondary education were more unlikely to use CAM than those with lower and no formal education. Findings from another study conducted by Amira et al. [26] in Lagos, Nigeria, showed that CAM was independent of all socio-demographic factors. In this work, sex is not significantly associated with the use of CAM. This is however not the situation in some other studies done in the developed countries where women were more likely to use CAM [15,27]. Other studies done in Nigeria however showed greater male gender use [17,19,22].

Limitations- The interviewer bias is a possible bias in this study and it was taken care of by using those who were not directly involved in the care of the patients in each unit to administer the questionnaire. Another potential bias is the inclusion of hospital patients who are ordinarily expected to give great importance to allopathic medicine over alternative medicine. This bias was reduced by enlightening the patients that alternative medicine also have its place in modern health care. No information was collected on the types of surgical pathologies that gave rise to the surgical treatment of the patients and this may possibly affect the prevalence of CAM use in the studied population. This is a single centre study thus it may be difficult to generalize the result to the whole surgical outpatient population in Nigeria. A multicenter study will be needed to further establish the findings in the future.

## 5 Conclusions

The result of this study has shown that the prevalence of CAM usage among surgical outpatients is quite high and the major determinants of its use are older age, belief about CAM, the patient's safety perception and their level of income.

## Ethics statement

Ethical approval was obtained from the Ethical Review Committee of LAUTECH Teaching Hospital, Ogbomoso (approval number- LTH/OGB/EC/2021/230). Written informed consent was also completed by each participant who agreed to participate in the study.

## Consent for publication

Authors have given the journal the consent for publication.

## Availability of data and materials

Data will be made available at request.

## CRediT authorship contribution statement

**Oluwatosin Stephen Ilori:** Writing – review & editing, Writing – original draft, Project administration, Investigation, Formal analysis, Methodology, Conceptualization. **Olawale Olakunlehin:** Writing – review & editing, Writing – original draft, Validation, Methodology, Formal analysis, Supervision. **Oluwatosin Ruth Ilori:** Writing – review & editing, Writing – original draft, Validation, Methodology, Formal analysis, Data Analysis. **Phillip Oluwatobi Awodutire:** Writing – review & editing, Writing – original draft, Methodology, Data analysis. **Olajumoke Shittu:** Writing – review & editing, Writing – original draft, Project administration, Investigation, Formal analysis. **Chidi Ugwuoke:** Writing – review & editing, Writing – original draft, Project administration, Investigation, Formal analysis.

## Declaration of competing interest

The authors declare that they have no known competing financial interests or personal relationships that could have appeared to influence the work reported in this paper.
