# Supplementary material for: Safety perception and determinants of complementary and alternative medicine usage among surgery out-patients in LAUTECH Teaching hospital, ogbomoso, Nigeria
Source: Heliyon. 2024 Jan 20;10(2):e24835. doi: 10.1016/j.heliyon.2024.e24835 (PMC10834812; doi:10.1016/j.heliyon.2024.e24835)
Supplement: Multimedia component 1 [file mmc1.docx]

**QUESTIONNAIRE**

**SOCIODEMOGRAPHICS**

1. Age-
2. Sex –a)Male b) Female
3. Marital status- a) Single b) Married c) Divorced
4. Tribe: a) Yoruba b) Igbo c) Hausa d)Others(specify)
5. Religion: a) Christianity b) Islam c) Traditionalist d) Others
6. Level of Education-a) No formal education b) Primary c) Secondary d) tertiary
7. Occupation- a)Unemployed b) Unskilled c) Semi skilled d) Skilled
8. Average monthly income: a)<10,000 b)10,000-50,000 c) 50,100-100,000 d) >100,000
9. Surgical subspecialty: a)Orthopaedic Surgery b) General Surgery c) Plastic Surgery

d) Urology

**TYPES OF CAM USED**

**Biological based**:

Herbal drugs
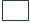
 High dose vitamins
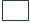


Kedi products
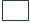
 medicinal tea
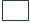
Forever living products
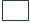


Urine therapy
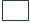
 Animal extracts (e.g python fat)
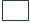
 Black stone
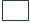
Others
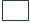


**Spiritual therapy**

Use of anointing oil
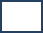
 Prayer
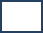
 Use of holy water
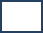
 Ritual sacrifice
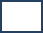


Divination/ incantation
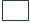
Others
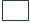


**Whole body therapy**

Acupuncture
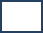
 Chiropractic
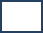
 Spinal manipulation
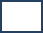
Others
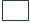


**Mind-Body Therapy**

Hypnosis
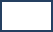
 Meditation
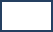
 Faith healing
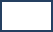
Others
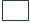


**Manipulative/ Body based therapy**

Massage
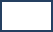
 Scarification
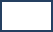
 Bloodletting
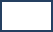
 Osteopathy/ Bone setting
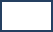


**PATTERN OF CAM USAGE**

1. Ever used CAM? Yes
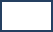
 No
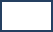

2. Current CAM use? Yes
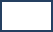
 No
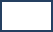

3. What symptoms or disease are you using CAM for?..............................................

**Reasons for exploring CAM: a)** Perceived limitation of conventional health care b) Dissatisfaction with pharmaceutical focus of conventional care c) Search for effective treatment for disease d) Positive personal experiences with CAM e) Others

**Reason for CAM usage: a)** Trust, affordability and availability b) Influence of family members and relations c) Incessant advertisement d) Others

**Reason for non-usage of CAM: a)** No necessity for CAM b) I don’t know about CAM possibility c) Doubt about its efficacy d) Afraid of side effects e) Too high cost f) others

**Pattern of CAM usage: a)** CAM only
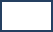
b)CAM with orthodox medicine
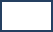
c)Orthodox medicine only
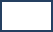


**Current CAM usage**: a) For current surgical complaint
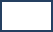
b) Others
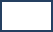


**Frequency of CAM use:**Daily
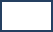
 Weekly
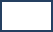
 Monthly
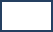
 Annually
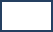
 Less than annually
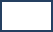


**Frequency of visit to CAM practitioner:**Daily
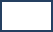
 weekly
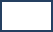
 Monthly
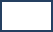
 Others
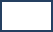


**Route of administration**:

Oral
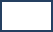
 per-rectal
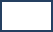
 skin application
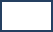
 per-vaginal
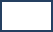
 nasal route
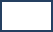
 via the eyes
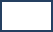
 via the ears
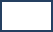
 others
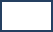


**BELIEF ABOUT CAM**

|  | **Agree** | **Undecided** | **Disagree** |
| --- | --- | --- | --- |
| CAM is very good in maintaining a healthy life |  |  |  |
| There are less side effects when taking CAM therapy |  |  |  |
| CAM use will increase if the government will develop it |  |  |  |
| CAM are healthier than taking conventional drugs |  |  |  |
| CAM enhances the body’s own defense mechanism and promotes self healing |  |  |  |
| CAM is good for the physical, mental and spiritual health |  |  |  |
| The more knowledge about CAM, the more the use |  |  |  |
| The more my friends use CAM, the more likely I use CAM. |  |  |  |
| CAM is more effective than orthodox medicine |  |  |  |
| The more the fears for conventional medicine, the more likely to use CAM |  |  |  |
| The people who do not have money are more likely to use CAM |  |  |  |

**SAFETY PERCEPTION OF CAM**

1. Safetyperception of CAM:Not sure
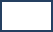
 Safe
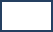
 Not safe
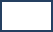

2. Safety concerns: Hygiene concerns
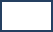
 Adverse effects
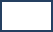
 lack of dosing regimen
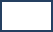
 content label
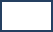

3. Concerns on causing side effects:Agree
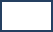
 Disagree
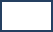
 Not sure
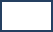

4. Record of side effects: Yes
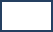
 No
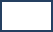
 Not sure
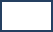

5. Record of interaction with orthodox drugs: Yes
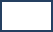
 No
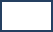
 Not Sure
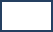

6. Concern on causing non compliance with orthodox medicine:Agree
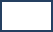
 Disagree
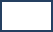
 Not sure
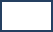


**Perceived adverse effect of CAM used**

1. Whether experienced adverse effect: a) Yes b) No
2. Specific perceived adverse effect: a) Cannot identify actual adverse effect b) Generalized body discomfort c) Dizziness d) Weakness e) Diarrhea f) Stomach upset e) Increase in body weight

**Perceived benefit from CAM use**

1. If benefitted: a)Yes b)No
2. Examples of CAM benefitted from: a) prayer b) Herbs c) Honey d) Massage e) others specify
3. Perceived CAM effectiveness: a)Effective with improved health status b) Not effective c) Cannot see any difference
